# 

**Published:** 1881-01

**Authors:** 


### Table of Contents,

### ORIGINAL COMMUNICATIONS.

Diphtheria.—A lecture. By Fiancis Delafield, M D., New York...... 577
Cancer, Trichinosis and Tuberculosis—Are they Incurable? By J. G. Westmoreland, M.D., Atlanta, Ga.................. 591

### REPORTS OF SOCIETIES.

New York Pathological Society.................. 595
Academy of Medicine.................. 604

### SELECTIONS.

On the Curability of Malignant Tumors of the breast by Adequate Operations. By Samuel W. Gross, A.M., M.D., Philadelphia...... 609
Placenta Previa. By J. P. Thomas, M.D.................. 614
Oxytocics in Obstetrics. By. S. R. Voorhees, M.D., Mason, Ohio... 624

### EDITORIAL.

Atlanta Hospital.................. 630
Cadaveric Alkaloids.................. 631
Meterological Report for December, 1880.................. 633

### BIBLIOGRAPHICAL.

Minor Surgical Gynæcology.................. 633

### EXCERPTA.

Infantile Constipation.................. 635
Multiple Sympathetic Affections Caused by the Presence of Intestinal Worms.................. 636
Ulcerating Surfaces and Abscesses.................. 637
Differential Diagnosis of Gastric Ulcer and Cancer.................. 638
Curious Case of Volitional Control over Involuntary Acts.................. 639
Treatment of Cystitis.................. 639
Action of Bromide of Potassium.................. 640
Liniment for Sore Nose.................. 640
Pilocarpin in Asthma.................. 640

